# Two-incision laparoscopic appendectomy for a severe hemophilia A child patient with coagulation factor VII deficiency: Case report and review of literature

**DOI:** 10.1097/MD.0000000000008197

**Published:** 2017-10-13

**Authors:** Jin Peng He, Jie Xiong Feng

**Affiliations:** Department of Pediatric Surgery, Tongji Hospital, Tongji Medical College, Huazhong University of Science and Technology, Wuhan, Hubei, China.

**Keywords:** appendectomy, child, coagulation factor VII deficiency, hemophilia A, laparoscopy

## Abstract

**Rationale:**

The main complication of patients with severe hemophilia is recurrent bleeding events that usually affected musculoskeletal contractures. And replacement therapy methods were continuously improved to minimize adverse impacts brought by those complications. However, only several cases reported about the appendectomy for hemophilia A. We report a case of acute appendicitis treated by two-incision laparoscopy in a boy with hemophilia A and coagulation factor VII deficiency for the first time.

**Patient concerns:**

An 8y7m-old Chinese boy presented with half a day of right sided abdominal pain, fever, nausea, and vomiting.

**Diagnoses:**

He received a computed tomography (CT) scan which revealed an enlarged appendix, thickened wall and appendiceal fecalith, and had received a conservative anti-bacterial treatment for his acute appendicitis but failed. He was diagnosed with hemophilia A and coagulation factor VII deficiency.

**Interventions:**

Two-incision laparoscopic appendectomy was made in success with a careful management of perioperative period. We monitored the clotting factor FVIII level and gave him a replacement therapy.

**Outcomes:**

The patient had an uneventful recovery.

**Lessons:**

It is important to exclude intraabdominal or retroperitoneal hemorrhage in patients suffering from hemophilia and acute abdominal pain. Pre-operative evaluation of validity of the FVIII replacement therapy is another effective strategy to assess the safety and feasibility of applying an operation procedure. The two-incision laparoscopic appendectomy is an effective treatment for this kind of patients for its minimal trauma and fast recovery characteristics. Our report shows that laparoscopic appendectomy is feasible in a child suffering from hemophilia after adequate blood clotting factor replacement treatment.

## 1 Introduction

Hemophilia is an inherited hematological system disorder caused by deficiency of factor VIII (hemophilia A) or factor IX (hemophilia B), which will lead to recurrent hemorrhages. There is around 1 in 5000 male births developed as hemophilia A.^[1]^ Systemic replacement with the deficient coagulation factors was developed as the basic treatment for hemophilia patients. While regular replacement therapy with clotting factor concentrates (CFCs) has completely transformed the lives of people with hemophilia over the past few decades, several challenges persist.

Acute appendicitis is 1 of the most common surgical emergencies in the pediatric population.^[2–4]^ However, the complaint is amphibious since no clear description of his/her symptoms is present. Therefore, a definite diagnosis is hard to make with a variety of clinical manifestations, especially difficulty to differentiate from gastroenteritis. Also, different surgical approaches such as the open appendectomy (OA), laparoscopic appendectomy (LA), and the transumbilical laparoscopic-assisted appendectomy were widely used in clinical approaches, but laparoscopic techniques usually were the best choice for minimal injury.^[5,6]^ But with severe hemophilia A, few reports discussed the treatment of acute appendicitis for this specific group of patients.^[7]^ Here, we describe a case of 2-incision LA in a child patient with severe hemophilia A and coagulation factor VII deficiency.

## 2 Patient history

**Figure 1:**
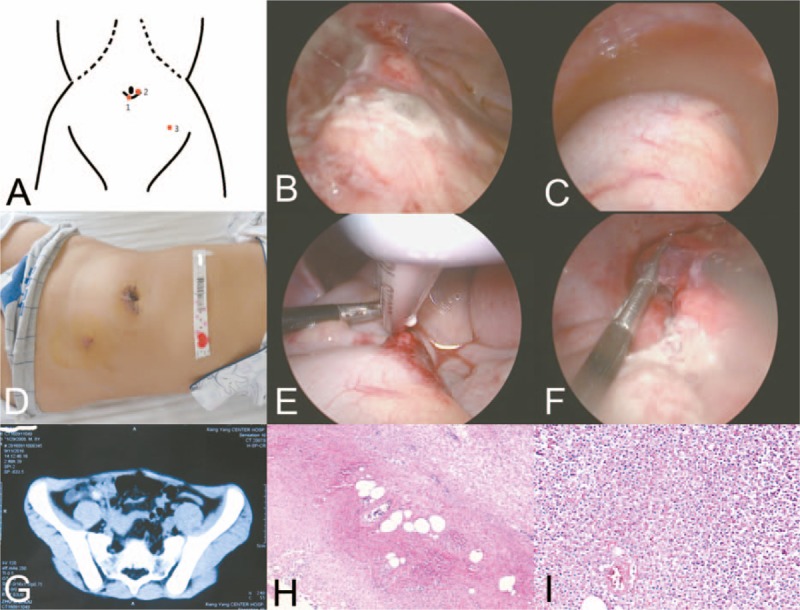
The figure of operation-correlated and examination. (A) The position of 3 trocars are shown with only 2 incisions. (B, C) Results of diagnostic laparoscopy, many exudates, and pus are seen in abdomen; the 2-incision wound healed, as shown in (D) showed. (B) and (C) indicates a clear view and minimal bleeding perioperation; a CT scan revealed an enlarged appendix, thickened wall, and appendiceal fecalith as shown in (G). (H, I) A pathological diagnosis of an acute phlegmonous appendicitis with periappendicitis is indicated.

An 8-year and 7-month old Chinese boy presented with half a day of right-sided abdominal pain, fever, nausea, and vomiting. He had an acute periumbilial pain that translated to the right-sided abdomen finally, with his body temperature rising up to 38.6°C. He was sent to the Xiangyang City Central Hospital and received a computed tomography (CT) scan which revealed an enlarged appendix, thickened wall, and appendiceal fecalith (Fig. 1G). However, his laboratory studies showed a hematocrit of 0.40, platelet count of 296 × 10^9^/L, white blood cell (WBC) count of 13.86 × 10^9^/L with differential count of 0.91 segmented neutrophils, 0.06 lymphocytes, and 0.02 monocytes. He was diagnosed with acute appendicitis and received antibacterial treatment (Cephalosporin), but the condition of the child deteriorated. Factor VIII activity level was unknown. His past medical history was significant for a mild hemophilia A without inhibitor. He had no previous surgeries and no such kind of family histories. He was transferred to Tongji Hospital, Wuhan, China, on day 2 of his illness. Examination revealed a mildly distended abdomen with hypoactive bowel sounds and fixed abdominal tenderness to palpation in the lower right quadrants without rebound or guarding. He received intravenous fluid and electrolyte supplements, 1 dose of antibacterial treatment (Cefoperazone), and 1 dose of antianaerobic bacteria treatment (Ornidazole). His abdomen pain deteriorated and laboratory studies revealed an acute exacerbation in the next 12 hours.

Exogenous and endogenous clotting factors were examined and revealed a documented factor VIII activity level of 1% and a documented factor VII activity level of 35% without inhibitor (titer <0.5 Bethesda units per milliliter [BU/mL]). The laboratory studies showed a hematocrit of 0.38, platelet count of 318.0 × 10^9^/L, WBC of 15.05 × 10^9^/L with differential count of 0.81 segmented neutrophils, 0.11 lymphocytes, and 0.08 monocytes. The C-reactive protein (CRP) rose up to 84.9 mg/L, but with normal serum amylase. Blood coagulation examination showed the following results: prothrombin time, 15.5 seconds (reference range 12.0–14.5 seconds); prothrombin time activity percentage, 70.0% (reference range 75.0%–125.0%); international normalized ratio, 1.27 (reference range 0.80–1.20); fibrinogen, 4.10 g/L (reference range 1.80–4.00 g/L); activated partial thromboplastin time (APTT), 95.6 seconds (reference range, 34.0–47.0 seconds); thrombin time, 15.4 seconds (reference range, 14.0–19.0 seconds).

## 3 Methods

### 3.1 Preoperative evaluation of validity of the FVIII replacement therapy

In view of the validity of the FVIII replacement therapy for this boy, 1500 IU recombinant coagulation factor VIII for injection (rFVIII, 50 IU/kg; Bayer HealthCare LLC) was injected and the factor VIII activity level was measured 2 hours later. The factor VIII activity level rose up to 72.0% (reference range, 60.0%–150.0%). Therefore, we considered the blood clotting factor substitution adequate in this child to undergo a major operation. A cooperative consultation with doctors from the hematology department approved our treatment strategy, and they suggested to inject 1 more dose of recombinant coagulation factor VII for injection (rFVII) if persistent bleeding was encountered during the operation. Also, another 1000 IU rFVIII was injected half an hour before the operation.

### 3.2 The 2-incision laparoscopic appendectomy

A 1-cm skin incision was first made below the umbilical margin and a 5-mm trocar was placed into the abdomen after incising the peritoneum (Fig. 1A and D). First, we explored the intestinal lesions by inserting the 5-mm, 30° optic scope through this port. Diagnostic laparoscopy was performed and revealed an ileocecal area adherent to the abdominal wall with signs of intra-abdominal infection. However, after lateral mobilization of the cecum, an abscess cavity was found (Fig. 1B and C). Another 5-mm trocar was then placed on the right side of the umbilicus. And another 5-mm trocar was then placed on the left lower quadrant (Fig. 1D). The appendix was then considered to be removed for a definite diagnosis of appendicitis (Fig. 1E and F). The stump of the appendix was closed by a ligating loop. We made a bacterial culture of the pus drained from the abscess around the appendix postoperatively. Informed consent was given to his parents.

## 4 Results

The boy underwent a 2-incision LA and rFVIII replacement treatment on day 2 of his illness. He received another 1500 IU rFVIII injection after operation. His APTT dropped into 47.2 seconds from 95.6 seconds. On days 3 to 5 of his illness, that is, days 1 to 3 after operation, the patient received 1500 IU rFVIII injection per 12 hours. His APTT dropped into 42.8 seconds from the 47.2 seconds, whereas his factor VIII activity level rose up to 141.0%. The urinary catheter was removed on day 4 of his illness and early ambulation was recommended immediately for him. There was about 50 mL intraperitoneal drainage presented as mixture of the blood and exudate during the first 2 days.

**Figure 2:**
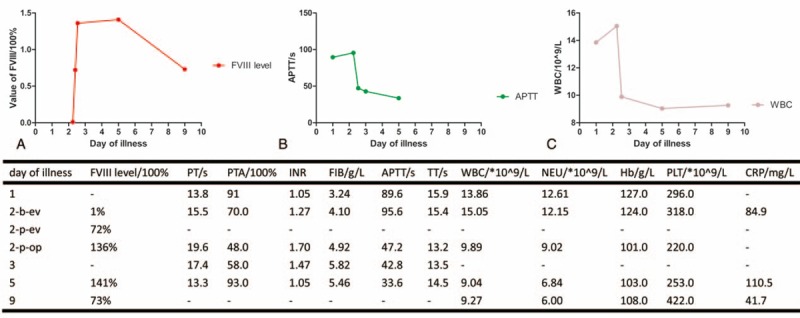
Monitoring of the factor VIII activity level and other laboratory findings. 2-b-ev = the day second of illness, but before the pre-evaluational injection of FVIII, 2-p-ev = the second day of illness, but after the pre-evaluational injection of FVIII, 2-p-op = the second day of illness, but after the operation, APTT = activated partial thromboplastin time, CRP = C-reactive protein, FIB = fibrinogen, FVIII = clotting factor VIII, Hb = hemoglobin, INR = international normalized ratio, NEU = neutrophil, PLT = platelet, PT = prothrombin time, PTA = prothrombin time activity percentage, TT = thrombin time, WBC = white blood count.

On days 6 to 9 of his illness, that is, day 4 to 7 after operation, the patient received 1000 IU rFVIII injection per 12 hours. The abdominal cavity drainage tube was removed for little intraperitoneal drainage on day 9 of his illness. On days 10 to 12 of his illness, that is, days 8 to 10 after operation, the patient received 500 IU rFVIII injection per 12 hours. He received intravenous fluid and electrolyte replacement, 1 dose of antibacterial treatment (Cefoperazone), and 1 dose of antianaerobic bacteria treatment (Ornidazole) per 12 hours after operation (Fig. 2). None of the bleeding tendency, fever, abdominal pain, and vomiting was recorded during the inpatient time. He was followed up for 3 months after operation and presented a good rehabilitation to normal life and back to school. The patient had an uneventful recovery.

Pathology of the removed appendix revealed the acute phlegmonous appendicitis with periappendicitis (Fig. 1H and I). Bacteriological results from abscess and peritoneal fluids were positive for *Escherichia coli* and *Pseudomonas aeruginosa*, which were both sensitive to Cefoperazone according to the antimicrobial susceptibility test.

## 5 Discussion

Musculoskeletal complication associated with recurrent bleeding events is the most common complaint for hemophilia patients, which led to the development of replacement therapy in controlling those complications. Some authors reported appendectomy in children suffering from hemophilia A.^[8,9]^ We report a case of acute appendicitis treated by 2-incision laparoscopy in a boy with hemophilia A and coagulation factor VII deficiency for the first time. It is quite a rare case for the child patient who suffered from not only hemophilia A, but combined with coagulation factor VII deficiency.

### 5.1 The diagnosis of appendicitis with hemophilia A

A diagnosis of hemophilia for a carrier's son is definite and easy to establish. A spontaneous bleeding such as painful swelling of the joints after a mild excise will provide a clue to suspect of hemophilia.^[1,10,11]^ In this case, his parents presented as normal. He had received a systemic rFVIII replacement treatment and a definite diagnosis was made in the past.

Laboratory examination of hemophilia patients usually revealed a prolonged APTT; however, a defect in the binding of factor VIII and von Willebrand factor should be excluded out before a clear diagnosis of hemophilia A.^[12–14]^ In this boy, the laboratory examination revealed an APTT of 95.6 seconds, nearly 2 times of the normal value, which is in conformity with his history. A detailed examination was made to confirm the diagnosis of hemophilia for this boy. Exogenous (clotting factor II, V, VII, X) and endogenous (clotting factor VIII, IX, XI, XII) clotting factors were examined and revealed with a documented factor VIII activity level of 1% and a documented factor VII activity level of 35% without inhibitor (titer <0.5 BU/mL).

There is a type classification for hemophilia A according to documented factor VIII activity level, that is, patients with severe hemophilia have a level of factor VIII <1%, patients with mild hemophilia have a level of factor VIII ranked from 1% to 5%, whereas patients with moderate hemophilia have a level of factor VIII ranked from 6% to 40%. The patients with hemophilia A usually develop an uncontrolled bleeding after minor trauma, dental procedures, or surgery. There is no history of trauma for him. However, the spontaneous bleeding should be suspected for his diagnosis of severe hemophilia A, especially with lack of factor VII for him. Taking the other symptoms such as fever and vomiting into consideration, it is prudent to determine immediately, whether there was an intra-abdominal bleeding in this case. Therefore, we checked his CT scan carefully and found no signs of hemorrhage, but an enlarged appendix, thickened wall, and appendiceal fecalith. We made the final diagnosis of appendicitis in combination with the blood count results and CRP.

However, hemorrhage must be considered as the most important differential diagnosis for those hemophilia patients when presented with acute abdominal pain.^[15–19]^ CT scan is quite important for this kind of patients in differentiation diagnosis, even though it cannot improve the prognosis of appendicitis in children.^[20–22]^ Additionally, the differential diagnosis included necrotizing fasciitis, gastrointestinal perforation, acute perforated appendicitis, or acute gastroenteritis. The experience of his case emphasizes the importance of correct diagnosis and careful management for an acute abdomen pain in hemophiliac patients.

In a word, the medical history offers an important clue to suspect a diagnosis of hemophilia, whereas the laboratory examination and CT scan provide a proof to make a differential diagnosis, especially to pay careful attention to APTT.

### 5.2 The pre-evaluation of replacement treatment

Hemophilia can be treated safely and effectively by infusion of CFCs that are currently available. However, we may meet an uncontrolled bleeding when using a replacement treatment for hemophilia A. What's the matter? Inhibitor development comes into a significant challenge during the treatment of hemophilia. If an inhibitor is developed, hemophilia patients can no longer be treated by replacement therapy. In summary, it is with high risk of uncontrolled bleeding and complications for those patients who develop an inhibitor. Therefore, it is important to test whether there is an inhibitor existing before replacement treatment.

After exposure to clotting factor replacement therapy, up to 30% of patients with severe hemophilia A (baseline FVIII activity <1% of normal) develop immunoglobulin G alloantibodies that bind to functional domains on the FVIII molecule and inhibit or neutralize its clotting function.^[23–25]^ Most FVIII alloantibodies (ie, inhibitors) develop early in life, with risk highest during the first 50 exposure days (EDs) to CFC and the majority occurring between 10 and 20 EDs.^[25,26]^

Inhibitors are evaluated by the Bethesda assay or a modification of this assay (ie, Nijmegen modification). Thus, we made a pre-evaluation of replacement treatment before the operation by the method in the above mentioned way. It is a convenient method to test the validity of replacement treatment immediately for this boy. Why we choose 2 hours past the injection of rFVIII as the test point of pre-evaluation? Generally speaking, the 2-incision LA will take us 2 hours to finish, and the half-time of the rFVIII is over 8 to 12 hours.^[27]^ We would like to carry out this operation during a known factor FVIII level, especially upper than 70%, which will offer us a clear operation view.

Therefore, inhibitors would be another point to focus on in the replacement of hemophilia A. The pre-evaluation of replacement treatment will let us know the validity of replacement treatment for every individual. We should keep in monitoring the inhibitors in case of uncontrolled bleeding when using a replacement treatment for hemophilia A.

### 5.3 The management of perioperative period

It is more feasible and safe for an appendicitis patient to receive a LA than an OA. Taking the wound infections, pain scores, and rehabilitation time into consideration, LA was proved to be a better choice. We choose a LA for this patient in consideration of minimizing the trauma to decrease the risk of bleeding and to make a detailed abdominal exploration to avoid omitting other probable causes. We applied a 2-incision instead of single-incision as to shorten the operation time and to ensure the surgery goes safely and smoothly.

If you fail to design a complete perioperative management plan, you prepare to fail this surgery for uncontrolled bleeding during operation. Factor VIII or factor IX concentrations should be raised to 80% to 100% immediately before surgery to provide a blood coagulation homeostasis during operation. Dynamic monitoring was also necessary and important. It is relatively appropriate to remain above 50% within 5 to 14 days after surgery.^[10,28]^ The factor VIII concentration was raised to 72.0% immediately before surgery and another dose of rFVIII (33 IU/kg, about 60% elevated) was adopted to supply the cost of FVIII during operation. Also, negligible bleeding occurred during the operation without more than 10 mL. None of the anesthetic accidents and tracheorrhagia happened. Down stair-step replacement treatment of the boy achieved success. The risk of bleeding decreased along with the time after operation. Down stair-step replacement treatment minimized the economic cost, while exerting greatest effect. Rehabilitation exercises were scheduled in early 4 hours after injection of the rFVIIII in a same principle.

Recombinant activated factor VII has been applied in treatment of hemophilia patients with inhibitors in recent time and has been shown to provide high efficacy rates. We prepared one dose of rFVIIa before operation but we didn’t use at last. rFVIIa^[29]^ or activated prothrombin complex concentrate (aPCC)^[30]^ was used in treatment of hemophilia patients with inhibitors, but was limited to be applied widely in clinical for their short half-lives (rFVIIa: 2.3–6.0 hours^[31–34]^; aPCC 4–7 hours; thrombin generation-based half-life).^[35]^ Therefore, new drugs need to be developed for replacement in the future, such as a recently developed medicine named as ACE910.^[36]^

## 6 Conclusions

In summary, factor VIII levels greater than 30% of the normal level are required for postoperative hemostasis. It is safe for a child patient with severe hemophilia A and coagulation factor VII deficiency to undergo laparoscopic appendicectomy if prepared carefully.

## Acknowledgments

We thank Dr Tian Qi Zhu and Ms Xiao Li Zhao for their help in care of this patient.
